# A New Perspective on Fault Geometry and Slip Distribution of the 2009 Dachaidan Mw 6.3 Earthquake from InSAR Observations

**DOI:** 10.3390/s150716786

**Published:** 2015-07-10

**Authors:** Yang Liu, Caijun Xu, Yangmao Wen, Hok Sum Fok

**Affiliations:** 1 School of Geodesy and Geomatics, Wuhan University, Wuhan 430079, China; E-Mails: cjxu@sgg.whu.edu.cn (C.X.); ymwen@sgg.whu.edu.cn (Y.W.); xshhuo@sgg.whu.edu.cn (H.S.F.); 2 Key Laboratory of Geospace Environment and Geodesy, Ministry of Education, Wuhan University, Wuhan 430079, China; 3 Collaborative Innovation Center for Geospatial Technology, Wuhan 430079, China

**Keywords:** InSAR, fault geometry, slip distribution, the 2009 Dachaidan Mw 6.3 earthquake, the Tibet Plateau, rupture propagation

## Abstract

On 28 August 2009, the northern margin of the Qaidam basin in the Tibet Plateau was ruptured by an Mw 6.3 earthquake. This study utilizes the Envisat ASAR images from descending Track 319 and ascending Track 455 for capturing the coseismic deformation resulting from this event, indicating that the earthquake fault rupture does not reach to the earth’s surface. We then propose a four-segmented fault model to investigate the coseismic deformation by determining the fault parameters, followed by inverting slip distribution. The preferred fault model shows that the rupture depths for all four fault planes mainly range from 2.0 km to 7.5 km, comparatively shallower than previous results up to ~13 km, and that the slip distribution on the fault plane is complex, exhibiting three slip peaks with a maximum of 2.44 m at a depth between 4.1 km and 4.9 km. The inverted geodetic moment is 3.85 × 10^18^ Nm (Mw 6.36). The 2009 event may rupture from the northwest to the southeast unilaterally, reaching the maximum at the central segment.

## 1. Introduction

**Figure 1:**
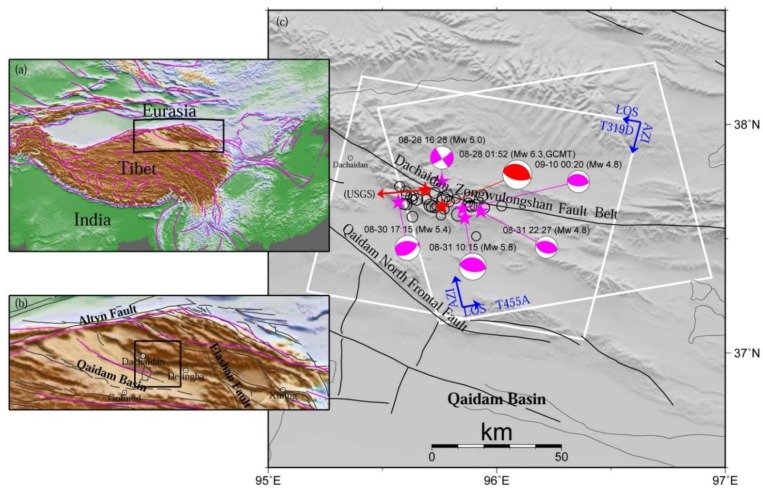
Regional tectonic setting of the 2009 Dachaidan Mw 6.3 earthquake. The rectangles in (**a**,**b**) are the spatial extents of (**b**,**c**), respectively. The white rectangles in (**c**) are the spatial extents of the Envisat ASAR descending Track 319 and ascending Track 455 images used in this study, with AZI and LOS referring to satellite azimuth and look direction, respectively. Thin black lines and purple lines in (**a**,**b**) denote the Quaternary active faults (Data from Deng *et al.* [3]), and the major tectonic faults (Data from Peltzer and Saucier [23]). A red beach ball with the time and magnitude labeled demonstrates the source mechanism for the main shock, while purple beach balls show the five aftershocks. Black hollow circles display the other aftershocks (Data from GCMT [2]). The epicenter of the main shock from USGS catalogue is also displayed.

At 01:52 on 28 August 2009 (UTC), an Mw 6.3 earthquake occurred at Dachaidan town, Haixi city, Qinghai province, China, which quaked as far away as Xining and Golmud cities in the Tibet Plateau (Figure 1) [1,2]. The seismogenic fault is located at the active fault belts of the northern Qaidam basin, where a number of active folds and faults striking North-West-West (NWW) direction can be clearly visualized. The fault belts are jointly intersected with the sinistral strike-slip Altyn fault system at the northwest side, and with the dextral strike-slip Elashan fault system at the southeast side, playing a significant role as a tectonic transition zone between these two strike-slip fault systems [3,4]. According to the Global Centroid Moment Tensor (GCMT) catalogue [2], five Mw ≥ 4.0 aftershocks occurred in the earthquake zone within an hour after the 2009 Mw 6.3 event, two of which had a moment magnitude of Mw 5.6 and occurred within half an hour, with a total of 34 Mw ≥ 4.0 aftershocks within one month (Figure 1). Before the 2009 main event, the other Mw 6.3 earthquake occurred almost at the same area on 10 November 2008 [5]. Determination of accurate source parameters for this main event is of the utmost importance for understanding local seismic risks and associated tectonic activities.

Rupture depth is of vital importance for assessing the static stress changes and the aftershock hazard in the surrounding regions during the postseismic phase, predicting the middle and larger earthquake location, and understanding the fault evolution. Using traditional seismological methods, the GCMT catalogue reported that this main event ruptured the fault plane with a strike of 101°, a dip of 60°, a rake of 83°, and a 12 km depth for the centroid (Table 1), whereas the rupture initiation depth for this event is 13 km when compared to the United States Geological Survey (USGS) catalogue [1]. Liu *et al.* [6] used the double-difference earthquake location algorithm and regional stations in Qinghai province to relocate the hypocenter parameters, obtaining a 6.5-km rupture initiation depth for this event, which is shallower than those from GCMT and USGS catalogues [1,2]. It can be revealed that this parameter for this main event differs from each other, ranging from 6.5 km to 13 km.

Recently, geodetic techniques have been employed for accurate monitoring of the surface deformation related to the earthquake fault [7]. The Interferometric Synthetic Aperture Radar (InSAR), a kind of geodetic measurement technique that does not require any ground control point, has frequently been utilized for analyzing the process, development, and occurrence of an earthquake in detail [8,9,10,11,12,13,14,15,16,17,18,19,20,21].

Using the above technique with two Envisat C-band interferogramic pairs, one ascending track and one descending track, Elliott *et al.* [5] extracted the coseismic deformation field with three peaks, arguing that the 2009 Dachaidan Mw 6.3 event consists of three separate fault ruptures (western, central, and eastern segments) dipping to the South-South-West (SSW) direction to model the observed deformation pattern. In addition, they suggested that the depth ranges of slip change significantly along the strike direction, which mainly occur from 3 km down to 11.8 km for the western segment, from 2.4 km down to 7.0 km for the central one, and from 1.9 km down to 6.6 km for the eastern one, respectively. However, we speculate that the difference in the inferred bottom rupture depth among these three segments is arguably large.

**Table 1:** Fault parameters of the 28 August 2009 Dachaidan Mw 6.3 earthquake and its five aftershocks.

		Longitude ^c^ (°)	Latitude ^c^ (°)	Strike (°)	Dip (°)	Length (km)	Top ^d^ (km)	Bottom ^d^ (km)	Rake (°)	Slip (m)	Moment ^e^ 10^18^ Nm	Mw
Western Segment ^a^	Fault 1	95.744	37.694	122.41	65.5	6.90	3.0	6.5	75.62	0.77	0.66	5.81
Central Segment ^a^	Fault 2	95.777	37.661	99.78	55.5	4.31	3.0	7.5	80.9	1.94	1.46	6.08
	Fault 3	95.844	37.652	99.78	55.5	6.07	3.0	7.0	85.85	0.70	0.66	5.85
Eastern Segment ^a^	Fault 4	95.940	37.646	104.26	46.0	6.15	2.0	6.0	101.24	0.73	0.80	5.90
	Average	95.826	37.663	106.56	55.6	23.43 ^f^	2.8	6.8	85.90	1.04	3.58 ^f^	6.34 ^f^
Mainshock ^b^	08-28 01:52	95.760	37.640	101	60	－	12.0 ^g^		83	－	3.0	6.3
Aftershocks ^b^	08-28 16:28	95.760	37.750	56	80	－	17.0^g^		−3	－	0.035	5.0
	08-30 17:15	95.570	37.660	272	45	－	16.8^g^		118	－	0.141	5.4
	08-31 10:15	95.860	37.590	98	57	－	12.0 ^g^		90	－	0.562	5.8
	08-31 22:27	95.930	37.620	107	45	－	12.0 ^g^		94	－	0.018	4.8
	09-10 00:20	95.850	37.630	91	55	－	15.7 ^g^		84	－	0.018	4.8

^a^ Western, central and eastern segments mean the extended fault 1, faults 2 and 3, fault 4, respectively; ^b^ Parameters from GCMT catalogue; ^c^ Center locations of the fault plane projected to the earth’s surface, and the centroid locations for GCMT parameters; ^d^ Top and bottom are the minimum and maximum depths of the fault plane, respectively; ^e^ Moment is calculated by assuming a shear modulus of 3.2 × 10^1^^0^ Pa; ^f^ Parameters are the sum of the corresponding parameters of four faults. Mw of 6.34 is estimated from the summed earthquake moment, 3.58 × 10^18^ Nm; ^g^ Depth of the centroid.

In this study, compared to Elliott *et al.* [5], we construct a fault model with four segments to investigate the coseismic deformation by determining the fault parameters using a dislocated model in a homogeneous half-space [22], in particular the rupture depth, by using the descending and ascending InSAR observations of the Dachaidan Mw 6.3 earthquake in 2009. To be more realistic for the rupture model, we further refine it by inverting InSAR observations for the slip distribution on the fault plane. We then discuss the rupture propagation pattern of the main event by jointly considering the results from GCMT, USGS, and this work. Finally, we also interpret the tectonic implications of the 2009 Dachaidan Mw 6.3 earthquake.

## 2. InSAR Data Processing and Coseismic Deformation

### 2.1. InSAR Data Processing

The InSAR method has become a widely used technique for extracting the deformation of the earth’s surface resulting from an earthquake event [8,9,10,11,12,13,14,15,16,17,18,19,20,21]. In this study, to map the 28 August 2009 Dachaidan Mw 6.3 earthquake displacement field, SAR images were acquired before and after the event by the Envisat satellite, which operates at C-band wavelengths (*i.e.*, 5.67 cm). Fortunately, both descending (Track 319) and ascending (Track 455) images can be used to produce the deformation interferograms (Figure 1; Table 2), which can provide two different viewing geometries to reduce the uncertainties of the inverted fault parameters [24,25]. Unfortunately, the ascending images only covered the eastern half of the deformation field. The perpendicular baselines of the descending and ascending interferometric pairs are 222.02 m and 180.3 m, respectively. Note the descending pair contains 19 days of postseismic deformation, while the ascending pair has 63 days (Table 2).

**Figure 2:**
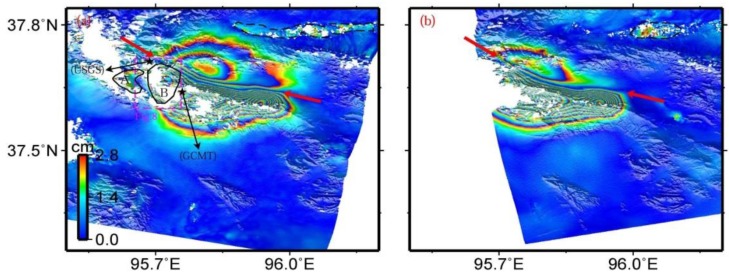
Interferograms of the descending track T319 (**a**) and the ascending track T455 (**b**) for the 2009 Dachaidan Mw 6.3 earthquake. Red arrows point out the deformation boundaries with a South-East-East (SEE) direction between hanging wall and footwall, which are used to determine the fault locations in the inversion step. The deformation zone labeled as A and delimited by the black polygon in (**a**), isolated from the deformation zone B, may result from atmospheric errors and/or aftershocks. The dashed purple rectangle in (**a**) is the spatial extent of Figure 8. The dashed black polygons in (**a**,**b**) delimit the zones that mainly include contribution from atmospheric errors, DEM errors, and other unknown errors. Epicenters of the main shock from GCMT and USGS catalogues are displayed in (**a**).

Both descending and ascending interferograms were produced from ASAR level 0 (raw data) images using the Caltech/JPL ROI_PAC software [26]. Topographic contributions were removed from the interferograms using the 3 arc-second Digital Elevation Model (DEM) produced by the Shuttle Radar Topography Mission (SRTM) [27]. Precise orbits from European Space Agency (ESA) were used for the orbital corrections. The differential interferograms were then filtered with the power spectrum filter technique [28] and unwrapped with the branch cut method [29]. The two interferometric deformation maps were finally geocoded to geographic coordinate system (Figure 2).

**Table 2:** SAR satellite data used in this study.

	Track	Date 1 (yyyymmdd)	Date 2 (yyyymmdd)	Days ^a^	B_perp_^b^ (m)	Σ ^c^ (cm)	*L* ^d^ (km)
Descending	319	20090114	20090916	19	222.02	0.52	3.84
Ascending	455	20090508	20091030	63	180.30	0.48	4.66

^a^ Time interval of days after earthquake; ^b^ Perpendicular baseline at the scene center; ^c^ Standard deviation of the interferogram’s noise; ^d^ e-folding spatial scale of the interferogram’s 1D covariance function, calculated using data from the interferogram, masked out within area of epicenter [30].

### 2.2. Coseismic Deformation

The surface deformation resulting from the Dachaidan Mw 6.3 earthquake can be clearly observed in both descending and ascending interferograms (Figure 2). Obvious deformation boundaries, with a South-East-East (SEE) direction between hanging wall and footwall, can be visually extracted in both descending and ascending interferograms (red arrows in Figure 2). These are used to determine the fault locations in the inversion step. There are continuous fringes across them, indicating that the earthquake fault did not rupture the earth’s surface. The deformation zones to the south of the boundary have denser fringes than those to the north of the boundary. This asymmetrical deformation pattern reveals that the earthquake fault is not vertical and should have south-dipping fault geometry. Both of these interferograms show a range decrease to the south of the boundary, and range increase on the opposite side. This phenomenon suggests that vertical motion dominates the surface deformation. These speculations are in line with one of the nodal planes of the GCMT focal mechanism (Figure 1 and Table 1). We note that the deformation zone (labeled as A in Figure 2a), isolated from the deformation zone B, may result from aftershocks and/or atmospheric errors presented in interferograms.

Some localized incoherence areas (e.g., blank areas around the main deformation zone and in the dashed black polygons in Figure 2) are evident in both descending and ascending interferograms, which may be related to the local vegetation conditions or variable atmospheric turbulence. The degree of incoherence in the descending interferogram is slightly larger than that in the ascending interferogram. Some of the possible reasons are that the perpendicular baseline for the descending interferogram is about 40 m larger than that for the ascending interferogram, or that the descending pair has a longer time span. In addition, the differences between the deformation patterns of the two interferograms should result from the different viewing geometries. For example, on the north of the deformation boundary, the descending interferogram yields about three fringes, whereas only about two fringes are apparent in the ascending interferogram.

An interferogram usually includes contributions from atmospheric path delays, orbital, DEM, and other unknown errors [30,31]. These factors affect the interferograms with different magnitudes and wavelengths. One-D covariance model, which has been widely used to investigate the magnitude and spatial wavelength of the interferogramic errors [32,33], is employed in this study to calculate the values of the corresponding parameters for each interferogram. The magnitude and spatial wavelength of errors are 0.52 cm and 3.84 km for the descending interferogram, and 0.48 cm and 4.66 km for the ascending interferogram (Table 2). In the inversion step, 100 datasets perturbed with noise of the statistical properties for each interferogram are generated with these parameters, followed by estimation of the standard deviation of slip distribution through inversion.

## 3. Inversion for Source Models

**Figure 3:**
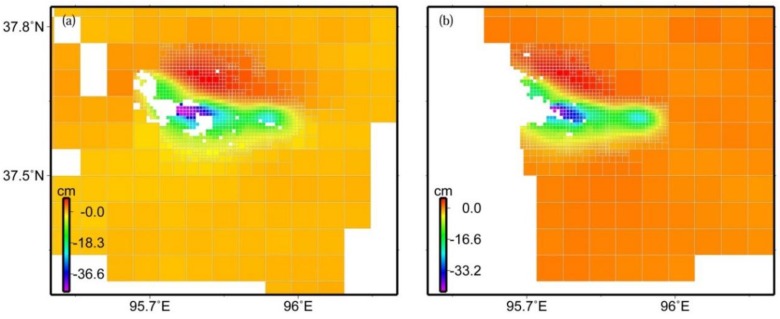
Down-sampled interferogram data of descending track 319 (**a**) and ascending track 455 (**b**) using the quadtree decomposition algorithm. Positive values indicate motion away from the satellite along its LOS, while negative values indicate motion toward the satellite.

We first use the InSAR data to determine the fault geometry with four segments, followed by solving for a more realistic slip distribution model. Before the inversion analysis, we resample the unwrapped interferometric phase (Figure 2) using the quadtree decomposition algorithm [34,35], a simple technique that is used to obtain an image representation at different resolution levels. For a given quadrant, if the variance is above a prescribed threshold (e.g., 9 mm^2^ in this study), the quadrant is then divided into four new quadrants. The process is iterated until either each quadrant has below-threshold variance, or until the quadrant reaches a minimum block size (e.g., 8 × 8 pixels in this study). After these processes, the numbers of the resampled interferogram data sets are reduced from 670,580 to 1229 for descending track 319, and from 517,910 to 1092 for ascending tack 455 (Figure 3). For each resampled observation point, variable Line-of-Sight (LOS) unit vectors are calculated precisely by considering observing geometry and topography. We also include an orbital ramp to model the residual orbit errors or long-wavelength atmospheric noise [25,33].

### 3.1. Fault Geometry Determination

To determine the fault geometry for the 2009 Dachaidan Mw 6.3 earthquake, we model InSAR observations with uniform slip on the rectangular dislocation in an elastic half-space using the equations in [22]. The elastic shear modulus and Poisson ratio are set to 3.2 × 10^10^ Pa and 0.25, respectively [35].

In the inversion process, we first derive the locations and strikes of the modeled fault directly according to the displacement boundaries in the coseismic surface deformation (Figure 2). The initial derived fault model consists of three segments whose strikes range from 99.78° to 122.41°, which are very close to those in Elliott *et al.* [5]. These three segments are not continuous. According to the inversion calculations, it is found that three independent segments cannot better interpret the observations. The reason for this may be that the differences of the slip distribution across the middle segment are too large to adopt only one segment to fit the surface observations, which is validated by the following slip distribution inversion in Section 3.2. The middle segment is then divided into two separate sub-segments, named faults 2 and 3, respectively (Table 1). In the following inversion, to reduce the number of the parameters to be estimated, the strikes and dips of faults 2 and 3 are assumed to be equal.

The dips and the top and bottom depths for the four segments are determined by the grid search method that best interprets the observations of the descending and ascending interferograms. For these four fault segments, a total of 12 parameters are required to be iteratively optimized. Because dips of faults 2 and 3 are constrained to have equal values, the parameters to be optimized reduce to 11. At each search step, only one parameter is optimized while the remaining 10 parameters are fixed. At the same time, the rakes, slips, and lengths for all four fault segments are estimated by the downhill simplex method [36]. The search step repeats itself with the updated optimal parameters until the fault parameters can best fit the InSAR observations.

**Figure 4:**
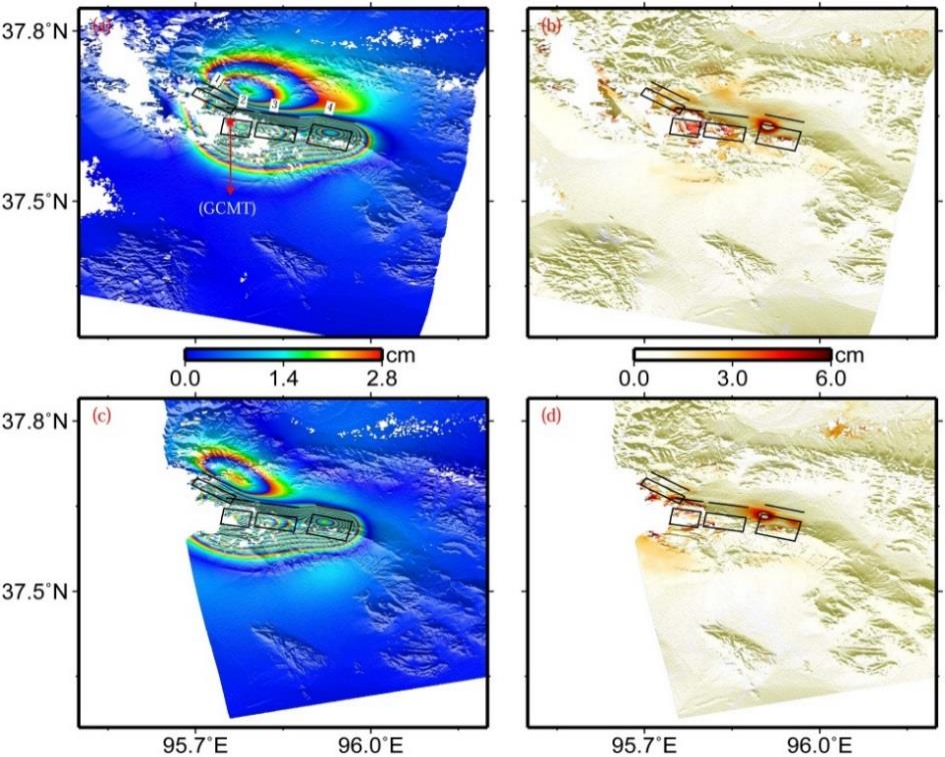
Modeled (**a**) and residual (**b**) interferograms predicted by uniform slip on four rectangular dislocations in an elastic half-space in the viewing geometry for the descending track T319, (**c**,**d**) for the ascending track T455. From the northwest to the southeast, four black rectangles denote the surface projections of faults 1, 2, 3, and 4 in Table 1, respectively, and black lines for the up-dip projections of the corresponding fault plane to the earth’s surface.

The preferred fault model solutions are shown in Table 1, and the surface projections of the four fault planes are shown in Figure 4. The estimated fault dips range from 46.0° to 65.5°, with an average dip of 55.6°. The fault planes are located at a depth of between 2.0 km and 7.5 km. This slip depth range is shallower than that from the GCMT catalogue, although the value in the GCMT catalogue refers to the centroid location. This can be attributable to different data sources providing different data constraints on the model parameters. The slip changes along the strike direction from northwest to southeast. The maximum slip of 1.94 m occurs at a depth range between 3.0 and 7.5 km on fault 2, the surface projection of which just covers the centroid location of this event from the GCMT catalogue (Figure 4a). The calculated geodetic moment for the four fault segments are 0.66 × 10^18^ Nm (Mw 5.81), 1.46 × 10^18^ Nm (Mw 6.08), 0.66 × 10^18^ Nm (Mw 5.85), and 0.80 × 10^18^ Nm (Mw 5.90), respectively, and the total geodetic moment is 3.58 × 10^18^ Nm (Mw 6.34).

The modeled interferograms and corresponding residual for the preferred fault model are shown in Figure 4. Both descending and ascending InSAR observations are generally well fitted by these four fault segments, despite some obvious residuals existing near the main deformation zone. These residuals potentially mainly result from the simplified fault model, the possible atmospheric propagation errors, DEM errors, and other unknown errors. The Root Mean Square (RMS) misfit for the descending and ascending interferograms are 0.87 cm and 0.85 cm, respectively, significantly lower than the result from Elliott *et al.* [5] (*i.e.*, 1.1 cm).

### 3.2. Slip Distribution Inversion

To obtain a more realistic rupture model, a refinement has been made by inverting InSAR observations for the slip distribution on the fault plane. In the inversion for the slip distribution, the geometry of the fault plane needs to be determined in advance [24,37]. For this purpose, we first fix the fault geometries (including strike, dip, and location) to the preferred solutions determined from the uniform slip modeling, and further extend the length and the top and bottom depths of each segment.

For the fault length, fault 1 is extended westward 1.1 km along the strike direction, and its final length is 8 km; fault 2 is first extended westward about 0.8 km along the strike direction, then extended eastward to connect with fault 3, finally continuing to the east about 2 km. The final length for the extended faults 2 and 3 are 14 km. Fault 4 is extended eastward 1.85 km along the strike direction, and its final length is 8 km. We refer to the extended fault 1, faults 2 and 3, and fault 4 as the western segment, the central segment, and the eastern segment, respectively (Table 1). In this way, the total length of this fault system is 30 km. For the top depth, all three segments are extended to the surface along the up-dip direction—that is to say, the top depths of them are entirely set to 0 km. For the bottom depth, the western, central, and eastern segments are extended to 7.28 km, 9.07 km, and 7.19 km, respectively. In this way, the widths for the western, central, and eastern segments are 8 km, 11 km, and 10 km, respectively.

**Figure 5:**
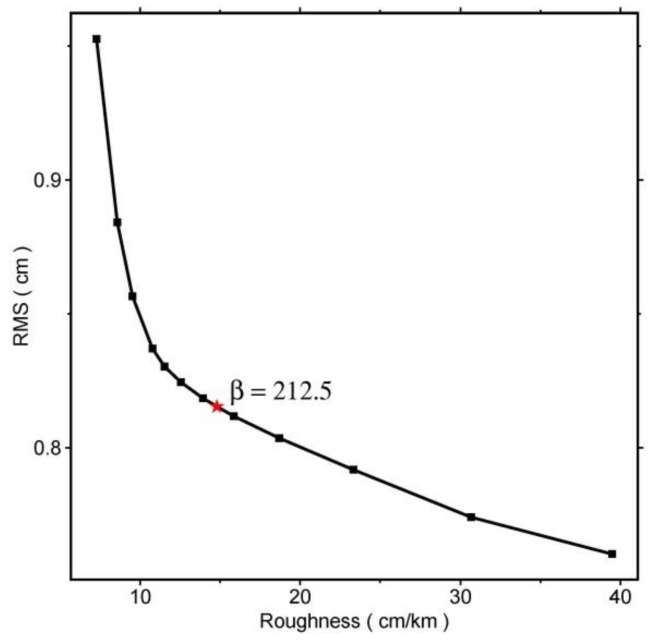
Trade-off curve between RMS misfit and the roughness. The red asterisk denotes the smooth factor chosen for the inversion.

The fault model was discretized into 298 patches, each with a length of 1 km and a width of 1 km. Consistent with the uniform slip inversion, dislocation equations in Okada [22] were used to model the InSAR surface displacement. Strike-slip and dip-slip components for each fault patch were solved in a least squares sense. Constraints of slip Laplacian smoothing across the fault patches were added to avoid unphysical oscillating slip distribution [38]. The smoothing factor (e.g., 212.5 in this study) was chosen by plotting the trade-off curve between RMS misfit and the solution roughness (Figure 5), which can obtain a slip distribution model that has both lower misfit and roughness.

**Figure 6:**
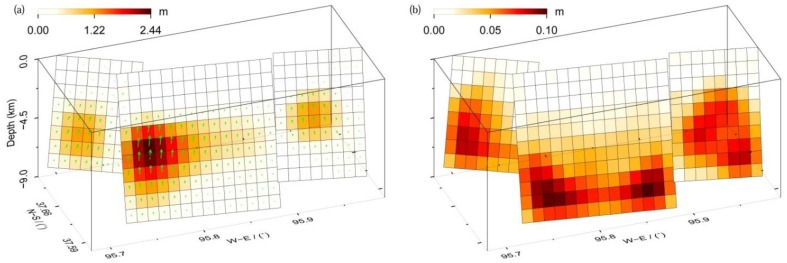
The preferred slip distribution (**a**) and its errors from Monte Carlo analysis (**b**). The green arrows in (**a**) denote the rake of each sub-fault.

**Figure 7:**
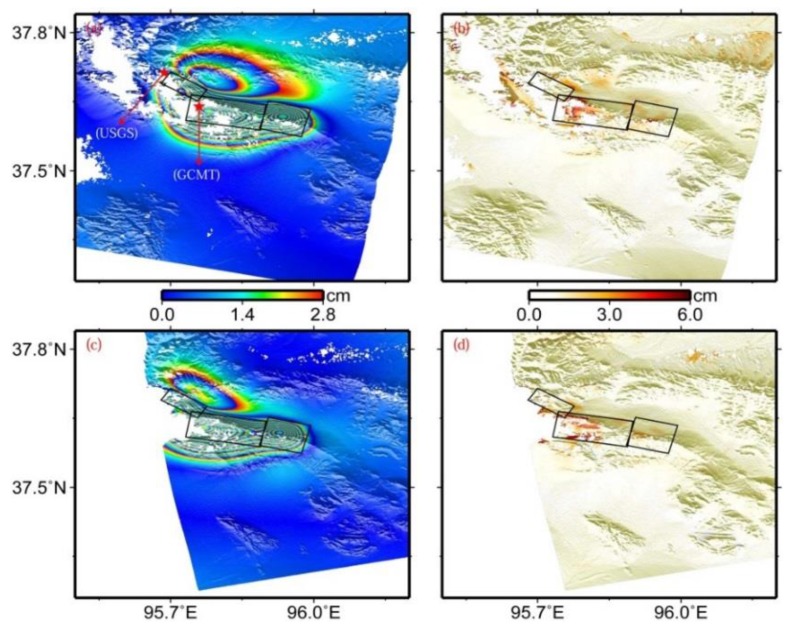
Modeled (**a**) and residual (**b**) interferograms predicted by the slip distribution model shown in Figure 6a in the viewing geometry for the descending track T319, (**c**) and (**d**) for the ascending track T455. From the northwest to the southeast, three black rectangles denote the surface projections of the western, central, and eastern segments mentioned in the text, respectively. Epicenters of the main shock from GCMT and USGS catalogues are displayed in subfigure (**a**).

Figure 6a shows the obtained slip distribution, with a total geodetic moment of 3.85 × 10^18^ Nm (Mw 6.36). The slip distribution apparently changes along the strike direction, and exhibits three slip peaks. The main slip is concentrated at a depth of 2.2–8.2 km, with average rake angle and slip of 69.9° and 0.82 m for the western segment, 82.8° and 1.14 m for the central segment, and 99.8° and 0.85 m for the eastern segment. These estimations are, overall, in agreement with those derived from uniform slip modeling. The maximum slip is 2.44 m at a depth between 4.1 km and 4.9 km, with a surface projection location close to the centroid location obtained from the GCMT catalogue (Figure 6 and Figure 7). We note here that the peak-slip location does not have to be the centroid location in the GCMT catalogue. The maximum slip in the top 1 km is just 4 cm, which is too small to be detected. This result is consistent with the absence of deformation fringe offset at the earth’s surface (Figure 2).

To determine the uncertainty in the slip model, 100 Monte Carlo simulations of noisy datasets were generated that have the same covariance characteristics as the original InSAR observations, followed by using these perturbed datasets to determine the slip errors through inversion [24,25]. The estimated slip errors are shown in Figure 6b. In general, larger errors are located in deep fault patches, and the maximum value is 10 cm. The magnitude of the slip is obviously larger than that of its error on the corresponding patches, indicating a reliable slip model (Figure 6a).

The modeled interferograms and corresponding residual for the preferred slip model are shown in Figure 7. When comparing them with Figure 4, derived from the uniform slip model, we found that the slip distribution model interprets patterns of both interferograms better. The RMS misfits for the descending and ascending interferograms decrease from 0.87 cm and 0.85 cm for the uniform slip modeling to 0.82 cm and 0.81 cm for the slip distribution modeling, representing about 5.8% and 4.7% in improvement, respectively. Although this model does not significantly reduce RMS misfit, we still argue that this model represents a more realistic slip distribution than the uniform slip model described in Section 3.1. In addition, the RMS misfits for the two interferograms are 0.3 cm and 0.33 cm larger than the standard deviation of the observed InSAR coseismic displacements, respectively.

The residual observed in Figure 7b,d and the larger RMS misfits may be potentially partly related to the inevitable disadvantage of the sampling time interval of the InSAR technique and the occurrence of the dozens of aftershocks, besides the possible atmospheric propagation errors, DEM errors, and other unknown errors. The interferograms used in this study contain 19 and 63 days of postseismic deformation (Table 2). Within this time period, at least 30 Mw ≥ 4.0 aftershocks occurred in the earthquake zone, and the moment magnitude of the 10 events is larger than Mw 5.0, including one event with moment magnitude up to Mw 5.9 [2]. As a consequence, the derived interferograms shown in Figure 2 inevitably include some deformation resulting from these aftershocks. In addition, according to the GCMT source parameters of five aftershocks shown in Table 1 and Figure 1, the source parameters of the two events are significantly different from those of the main event. In the slip distribution inversion, however, only three fault planes are used to interpret the InSAR observations. These facts may partly provide an explanation for the visible fitting residuals existing in Figure 7b,d, and for the RMS misfits larger than the error level in the InSAR observations.

## 4. Discussions

### 4.1. Rupture Depth of the 2009 Mw 6.3 Event

The 2009 Dachaidan Mw 6.3 earthquake mainly ruptured four fault segments (Table 1). On faults 1, 2, 3, and 4, the rupture depths ranged from 3.0 km to 6.5 km, from 3.0 km to 7.5 km, from 3.0 km to 7.0 km, and from 2.0 km to 6.0 km, respectively. The distribution of depth range along the strike direction from northwest to southeast indicates that the bottom depths of faults 2 and 3 are ~1 km greater than those of faults 1 and 4.

Comparing with the results in Elliott *et al.* [5], the bottom depths of faults 2, 3, and 4 are, overall, in agreement with those of the central and eastern segments in Elliott *et al.* [5], but the bottom depth (6.5 km) of fault 1 is significantly shallower than that of the western segment in Elliott *et al.* [5]. The distinct differences can be attributed to the available InSAR observations and the inversion method. Both descending and ascending observations from this study and Elliott *et al.* [5] show that the zone corresponding to the surface projection of fault 1 provides fewer observations due to data incoherence and/or observing geometry, leading to a looser constraint on the corresponding parameter estimations.

**Figure 8:**
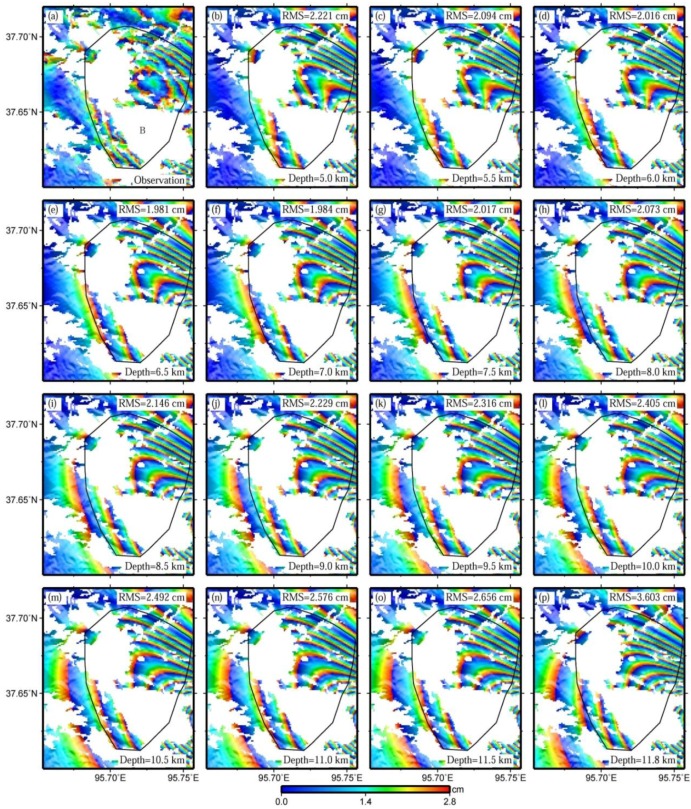
Observed interferogram (**a**), predicted ones with the bottom depths of fault 1 ranging from 5 km to 11.5 km at a step of 0.5 km (**b–o**), and the predicted one with the corresponding parameters equal to those in Elliott *et al.* [5] (**p**). The spatial extent of these figures is shown in Figure 2. Note that the RMS errors of sub-figures (**b**–**p**) are calculated with observations located in and around the black polygons.

In the uniform slip inversion of Elliott *et al.* [5], the central segment is assumed to be a uniform slip of 1.0 m, and not divided into two separate sub-segments. The inversion results with a four-segmented fault model in this study indicate, however, that the slip differs significantly across the central segment, changing from 1.94 m on fault 2 to 0.7 m on fault 3 (Table 1). We speculate that the slip for the western part of the central segment in Elliott *et al.* [5] might be underestimated. As a consequence, a deeper bottom depth of the central segment in Elliott *et al.* [5] would probably be required to better interpret the InSAR observations.

An experiment was conducted by varying the bottom depths of fault 1, ranging from 5 km to 11.5 km at a step of 0.5 km (Figure 8b–o), revealing that a bottom depth of 6.5 km shows optimal fitting for the observed pattern. When the bottom depth of fault 1 reduces from 6.5 km to 5 km, the lower-left region of the deformation zone B in Figure 8a is actually narrowed by the predicted deformation (Figure 8b–d). However, it is broadened when the bottom depth of fault 1 increases from 6.5 km to 11.5 km (Figure 8f–o). This broadening shows consistency with the results obtained by Elliott *et al.* [5] (Figure 8p).

Further, we compare the results from uniform slip modeling and slip distribution modeling. According to the slip distribution inversion results, the main slip is concentrated at a depth between 2.73 km and 7.28 km for the western segment, between 2.47 km and 8.24 km for the central segment, and between 2.16 km and 5.75 km for the eastern segment. It is apparent that these estimations are in agreement with those derived from uniform slip modeling.

We then compare our preferred rupture depth with the results from the traditional seismological studies. The rupture depth for all four fault planes, ranging from 2.0 km to 7.5 km, is obviously shallower than that from GCMT and USGS catalogues, with the depths of the centroid location and the rupture initiation of 12 km and 13 km, respectively. However, the derived depth range is consistent with that (6.5 km) from Liu *et al.* [6], in which relocations of the 2009 Dachaidan Mw 6.3 earthquake and its aftershocks recorded by three or more stations of the regional seismic network center of Qinghai province were determined using the double-difference earthquake location method.

### 4.2. Rupture Propagation of the 2009 Mw 6.3 Event

Figure 7a shows the epicenters from the GCMT and USGS catalogues [1,2]. The epicenter from the GCMT catalogue is within the surface projection of the central fault plane with a maximum slip of 2.44 m, whereas the one from the USGS catalogue lies close to the northwest end of the surface projection of the western fault plane. It is common knowledge that the epicenter from the GCMT catalogue is the location of the earthquake centroid, and the one from the USGS catalogue is the initiation point of the earthquake rupture. According to Figure 7a, it was found that the location from USGS is northwest of that from GCMT. Provided that these results are reliable, one can infer that this main event ruptures unilaterally from the northwest to the southeast, and reaches a maximum slip at the central segment.

The preferred fault model (Table 1 and Figure 7) shows that the strikes for this event change significantly from northwest to southeast, with values of 122.41°, 99.78°, and 104.26° for the western, central, and eastern segments, respectively. The transition areas among these three fault segments can be seen as two step-overs, though no obvious surface breaks are observed in this earthquake. The spatial distance of the step-over between the western and central segments is about 2 km, and less than 1 km for the other step-over. Together with the slip distribution in Figure 6a, this might suggest that the step-overs are responsible for the rupture propagation during the 2009 earthquake, which has also been observed in the 2001 Kokoxili Mw 7.9 earthquake and the 2008 Wenchuan Mw 7.9 earthquake occurring at the Tibet Plateau [32,39]. Besides, both uniform slip and slip distribution inversions indicate that the average rake angles change from ~70° on the western segments, to ~80° on the central segment, and to ~100° on the eastern segment. These indicate that the fault strike influenced the extension of the slip vector of the earthquake rupture fault in the 2009 Dachaidan Mw 6.3 event.

### 4.3. Tectonic Implications of the 2009 Mw 6.3 Event

The 2009 Dachaidan Mw 6.3 earthquake occurred on the Dachaidan–Zongwulongshan fault belt, located at the northern edge of the huge sedimentary Qaidam basin [1,2,40]. This zone has experienced significant tectonic activity since the Indosinian movement in the late Triassic period [41,42]. Based on the lithology, gravity, magnetic field, magnetotelluric depth data, and other existing data, Chen *et al.* [41] argued that the basement tectonic systems in the pre-Mesozoic strata of the Qaidam basin have controlled the development and distribution of the sedimentary cover and its internal fault belts. The estimated basement depth around the Qaidam basin is about 5–13 km using aeromagnetic data from Xiong *et al.* [43].

Before the 2009 Mw 6.3 event, on 10 November 2008, another Mw 6.3 earthquake occurred almost at the same area, the epicenter of which was less than 8 km away from that of the 2009 event. Using InSAR observations to invert the fault rupture parameters of the 2008 event, Elliott *et al.* [5] suggested that the earthquake fault mainly ruptured a depth ranging from 11.3 km to 21.6 km, with a strike and dip of 99° and 67°. Further, Elliott *et al.* [5] argued that the 2008 event triggered the 2009 event, according to the Coulomb stress calculation. In this study, our preferred fault model shows that the depth intervals of slip mainly range from 2.0 km down to 7.5 km, and the average strike and dip are 106.6° and 55.6°. The strikes and dips for the two events are mutually consistent, but the rupture zone of the 2008 event is below that of the 2009 event along the depth direction.

Further, provided that the basement depth around the Qaidam basin and the rupture depths of the 2008 and 2009 events mentioned above are reliable, one can infer that the 2008 event may have occurred in the basement rock at a depth of ~11–22 km, and the 2009 event may have occurred in the covered rock at a depth of ~2–7.5 km. This indicates that the two events ruptured in two tectonic layers, one at the basement tectonic layer and the other at the covered tectonic layer. Further, if the 2008 event occurring in the basement layer indeed triggered the 2009 event occurring in the covered layer, we speculate that the temporal and spatial characteristics of the 2008 and 2009 events may potentially provide evidence for the role of basement tectonic systems in controlling the development and distribution of covered fault belts around the 2009 Dachaidan Mw 6.3 earthquake [41].

## 5. Conclusions

This study uses InSAR observations to map the coseismic displacement field and to investigate the fault rupture characteristics related to the 28 August 2009 Dachaidan Mw 6.3 earthquake, occurring at the northern margin of the Qaidam basin in the Tibet Plateau. The deformation patterns observed by both descending and ascending interferograms indicate that the earthquake event had complex fault geometry, and did not rupture the earth’s surface.

The preferred fault geometry with four segments suggests that the strikes for this event changed significantly from northwest to southeast, with values of 122.41°, 99.78°, and 104.26° for the western (fault 1), central (faults 2 and 3), and eastern (fault 4) segments, respectively. The rupture depths for all four fault planes mainly range between 2.0 km and 7.5 km, comparatively shallower than previous results up to ~13 km. The preferred slip distribution model exhibits three slip peaks, with maximum slip of 2.44 m at a depth of 4.1–4.9 km, and the total geodetic moment is 3.85 × 10^18^ Nm (Mw 6.36).

Combined with the GCMT and USGS catalogues, the inversion results indicate that the 2009 event expresses unilateral rupture from the northwest to the southeast, with a maximum slip at the central segment. In addition, the findings may potentially provide an evidence for the role of the basement tectonic systems controlling the development and distribution of the covered fault belts around the 2009 Dachaidan Mw 6.3 earthquake.
